# Nanomaterials for Tumor Hypoxia Relief to Improve the Efficacy of ROS-Generated Cancer Therapy

**DOI:** 10.3389/fchem.2021.649158

**Published:** 2021-03-19

**Authors:** Changping Ruan, Kaihua Su, Dongmin Zhao, Ai Lu, Chaoran Zhong

**Affiliations:** State Key Laboratory for Chemistry and Molecular Engineering of Medicinal Resources, School of Chemistry and Pharmaceutical Sciences, Guangxi Normal University, Guilin, China

**Keywords:** reactive oxygen species, tumor hypoxia, cancer therapy, nanomaterials, O_2_ supply, tumor oxygenation

## Abstract

Given the fact that excessive levels of reactive oxygen species (ROS) induce damage to proteins, lipids, and DNA, various ROS-generating agents and strategies have been explored to induce cell death and tumor destruction by generating ROS above toxic threshold. Unfortunately, hypoxia in tumor microenvironment (TME) not only promotes tumor metastasis but also enhances tumor resistance to the ROS-generated cancer therapies, thus leading to ineffective therapeutic outcomes. A variety of nanotechnology-based approaches that generate or release O_2_ continuously to overcome hypoxia in TME have showed promising results to improve the efficacy of ROS-generated cancer therapy. In this minireview, we present an overview of current nanomaterial-based strategies for advanced cancer therapy by modulating the hypoxia in the TME and promoting ROS generation. Particular emphasis is put on the O_2_ supply capability and mechanism of these nanoplatforms. Future challenges and opportunities of design consideration are also discussed. We believe that this review may provide some useful inspiration for the design and construction of other advanced nanomaterials with O_2_ supply ability for overcoming the tumor hypoxia-associated resistance of ROS-mediated cancer therapy and thus promoting ROS-generated cancer therapeutics.

## Introduction

ROS (including singlet oxygen (^1^O_2_), superoxide radicals (O_2_^•−^), hydroxyl radicals (•OH), and peroxides (O_2_^2−^)) play a concentration-dependent role in physiological activity (Gorrini et al., 2013). Low to moderate levels of ROS regulate cell signaling and promote cell proliferation, and elevated levels of cellular ROS are one of the unique characteristics of cancer, whereas excessive ROS will induce nonspecific damage to proteins, lipids, and DNA. Because of the heightened basal level of ROS in cancer cells, cancer cells are more susceptible to exogenous ROS, compared to normal cells that maintain redox homeostasis (Yang B. et al., 2019). Therefore, modulation of the ROS level at cancer cells has been emerging as promising strategy for the tumor destruction by generating ROS above toxic threshold. Hypoxia, mild acid, and overexpressed H_2_O_2_ are three characteristic features of tumor microenvironment (TME) (Dai et al., 2017; Kwon et al., 2019). Because of the aggressive proliferation of cancer cells and the insufficient blood supply in tumors, the O_2_ supply in solid tumors was usually insufficient (partial pressure of O_2_ < 2.5 mmHg). Hypoxia in TME not only promotes tumor metastasis but also enhances tumor resistance to the ROS-generated cancer therapies, such as photodynamic therapy (PDT), radiation therapy (RT), chemotherapy, chemodynamic therapy (CDT), and sonodynamic therapy (SDT), thus leading to ineffective therapeutic outcomes. Tumor oxygenation that aims at greatly increasing the oxygen concentrations in hypoxic tumors has been demonstrated to be an effective strategy to overcome tumor hypoxia and enhance the sensibility of hypoxic tumors toward the ROS-generated cancer therapy (Li et al., 2018; Yang B. et al., 2019).

To relieve hypoxia, hyperbaric oxygen therapy, which involves the breath of pure O_2_ in a pressurized chamber, has been developed. Unfortunately, its extensive application is limited by the intrinsic side effects including hyperoxic seizures and barotrauma as a result of the overproduced ROS in normal tissues (Kim et al., 2017). Also, angiogenesis inhibitors have been applied to transiently normalize the tumor vasculatures and suppress the consumption of O_2_. However, the oxygenation improvement resulting from the normalization of vessels only lasted for a few days (Liu J. N. et al., 2017). Promoted by recent advances in nanotechnology, a variety of nanotechnology-based approaches that generate or release O_2_ continuously to overcome hypoxia in TME have showed promising results to improve the efficacy of ROS-generated cancer therapy. In this minireview, we present an overview of current nanotechnology-based strategies for advanced cancer therapy by modulating the hypoxia in TME and promoting the generation of ROS. To amplify the therapeutic outcomes, the approach of modulating tumor hypoxia was usually applied in combination with other therapeutic/theranostic modalities. This minireview mainly focused on the O_2_ supply ability and mechanism of these nanoplatforms. Future challenges and opportunities of design consideration are also discussed and summarized.

### Tumor Hypoxia-Regulating Approaches Based on Nanotechnology

Based on their different mechanisms and involved materials, nanotechnology-based tumor hypoxia-regulating approaches can be classified into the following categories: delivering O_2_ by natural or artificial oxygen-carrying materials, the hydrolysis of exogenous peroxide, catalytic decomposition of intracellular H_2_O_2_ by utilizing catalase or catalase-like nanozymes, and generating O_2_ by water-splitting photocatalysts.

### Delivering O_2_ by Natural and Artificial Oxygen-Carrying Materials

**FIGURE 1:**
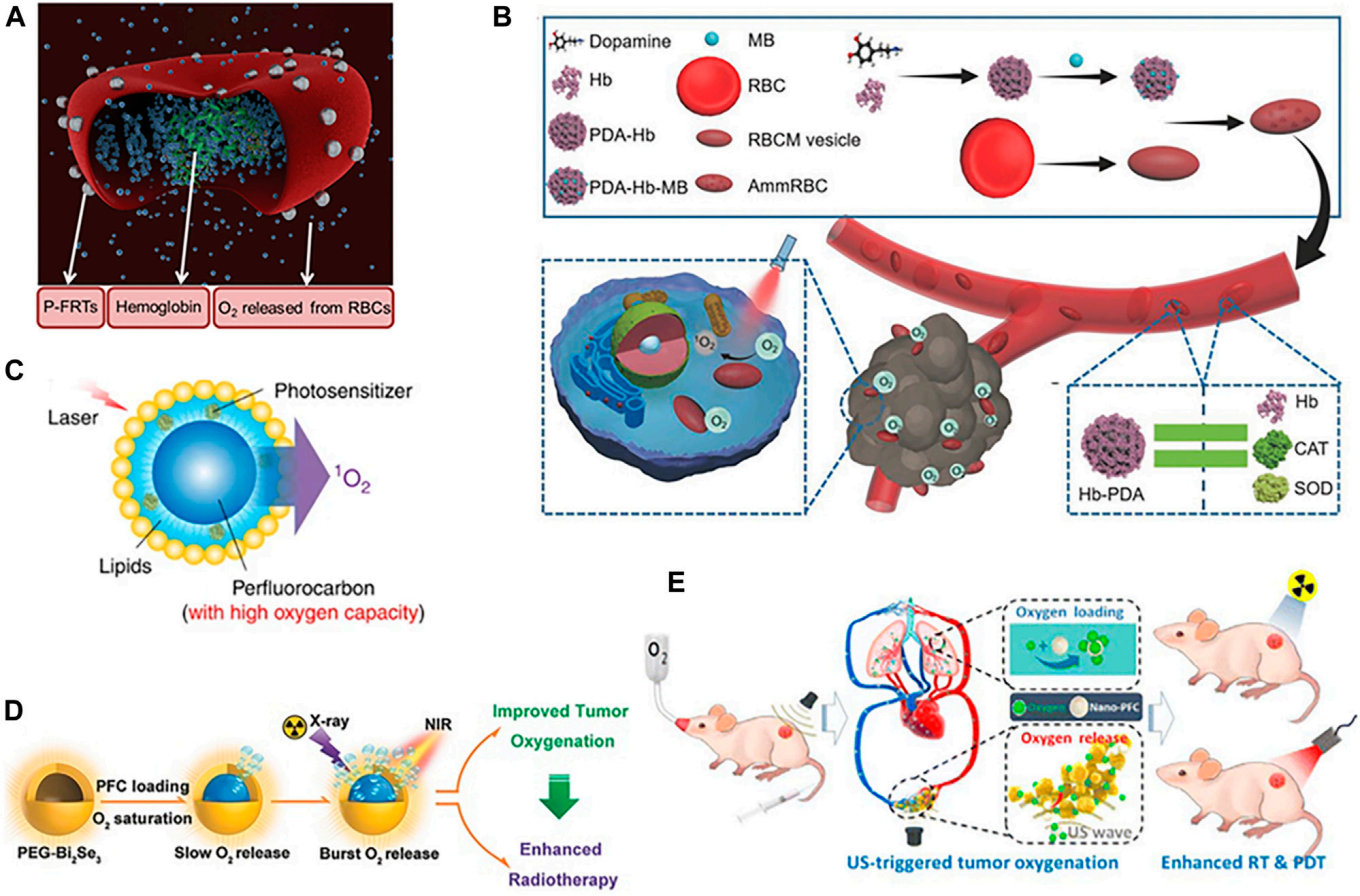
**(A)** Schematic illustration of the formation and working mechanism of P-FRT-RBCs (Tang et al., 2016) (Copyright 2016, reproduced with permission from John Wiley and Sons). **(B)** Schematic illustration of AmmRBCs that accumulate in the tumor site and boost ^1^O_2_ generation for enhanced PDT. Polydopamine (PDA) in AmmRBC functions like CAT and superoxide dismutase (SOD) in RBCs to protect Hb from oxidant damage during the circulation (Liu W. L. et al., 2018) (Copyright 2018, reproduced with permission from John Wiley and Sons). **(C)** Schematic illustration of the structure and design of the Oxy-PDT agent. Photosensitizer and perfluorocarbon are coencapsulated by lipids. Photosensitizers are uniformly dispersed inside the lipid monolayer and PFC in the core of the nanoparticle. When irradiated by laser, photosensitizer (PS) transfers energy to the oxygen enriched in PFC, producing ^1^O_2_ (Cheng et al., 2015) (Copyright 2015, reproduced with permission from Nature Publishing Group). **(D)** Schematic illustration of hollow PEG-Bi_2_Se_3_ nanoparticles with PFC loading as an oxygen carrier and the burst release of oxygen under stimulation by a NIR laser (Song G. S., Liang C. et al., 2016) (Copyright 2016, reproduced with permission from John Wiley and Sons). **(E)** Schematic illustration of the mechanism of US-triggered local oxygenation in the tumor using nano-PFC as the oxygen shuttle (Song X. J. et al., 2016) (Copyright 2016, reproduced with permission from American Chemical Society).

Red blood cells (RBCs), the primary source of O_2_ in mammals, contain 270 million hemoglobin (Hb) molecules per cell; each Hb molecule binds up to four O_2_. Hb allows efficient binding of O_2_ under high O_2_ pressure and rapid O_2_ release under hypoxic environment. Because of the good biocompatibility and long circulation, RBCs have been widely investigated as biological drug carriers and O_2_ shuttles for cancer therapy (Squires, 2002; Wang et al., 2013; Wang et al., 2014; Sun et al., 2015; Wang et al., 2017). Tang et al. (2016) demonstrated that RBCs tethered with photosensitizers (ZnF_16_Pc) onto the RBCs surface (P-FRT-RBCs) could realize the codelivery of O_2_ and photosensitizers (Figure 1A). The sustained O_2_ supply adjacent to photosensitizers by RBCs enabled efficient PDT even under hypoxic conditions. However, the micrometer sizes of RBCs may limit their extravascular diffusion ability and reduce their chance to approach tumor cell. The oxygen-carrying ability of RBCs is limited by the inherent oxygen-binding ability of Hb. However, cell-free Hb suffers from severe problems, including short circulation time, potential side effect, and poor stability. Hb-based O_2_ carriers via chemical modification or encapsulation with biodegradable materials could overcome the disadvantages of cell-free Hb and demonstrate the similar oxygen-carrying capability as that of natural RBCs (Gundersen and Palmer, 2008; Duan et al., 2012; Jia et al., 2012; Paciello et al., 2016; Zhou et al., 2016; Cao et al., 2018; Jansman and Hosta-Rigau, 2018; Yu et al., 2018; Hu et al., 2020). Compared to RBCs with micrometer sizes, nanodimensional Hb-based O_2_ carriers can perfuse tumor tissues within the narrow vascular structure and thus can supply more O_2_ in hypoxic tumor (Jia et al., 2016; Luo et al., 2016; Zhao et al., 2016). Inspired by the biological nature of RBCs, Liu W. L. et al. (2018) developed an aggressive man-made RBC (AmmRBC) as oxygen self-supplied PDT system to combat the hypoxia-mediated resistance of tumors to PDT (Figure 1B). This biomimetic platform was prepared by encapsulating methylene blue (MB) adsorbed Hb-polydopamine complex into the biovesicle engineered from the recombined RBC membranes. Polydopamine played the role of the antioxidative enzymes to prevent Hb from the oxidation damage during the circulation.

In recent years, an artificial blood product, perfluorocarbon (PFC) compounds with good biocompatibility and high oxygen dissolving ability, has been extensively used as O_2_ carriers to modulate the hypoxic TME (Squires, 2002; Lee et al., 2015; Que et al., 2016; Liang et al., 2020). By loading a near-infrared photosensitizer (IR780) into PFCs nanodroplets, Cheng et al. (2015) developed an oxygen self-enriching PDT (Oxy-PDT) nanoplatform (Figure 1C). Owing to the higher oxygen capacity and longer ^1^O_2_ lifetime of PFCs, the PDT effect of the loaded photosensitizer was significantly enhanced. Gao et al. (2017) reported erythrocyte-membrane coated PFC nanoparticles as artificial RBCs to deliver O_2_ and enhance radiation response.

Though having high oxygen solubility, PFC releases O_2_ simply by diffusion through the O_2_ concentration gradient, usually resulting in a low delivery efficiency. Using near-infrared (NIR) light or ultrasound (US) as trigger could accelerate the release of O_2_ and promote the tumor oxygenation (Song G. S., Liang C. et al., 2016; Chen et al., 2017). Song et al. utilized the photothermal effect of Bi_2_Se_3_ induced by NIR laser irradiation to trigger the burst release of O_2_ from PFC loaded inside the hollow Bi_2_Se_3_ nanoparticles, thereby greatly promoting the tumor oxygenation and overcoming the hypoxia-associated radioresistance of tumors (Song G. S., Liang C. et al., 2016) (Figure 1D). Song X. J. et al. (2016) used an external low-frequency/low-power US treatment to trigger the release of O_2_ from nano-PFC to relief tumor hypoxia for enhanced PDT and RT (Figure 1E). Given that several formulations of PFC emulsions have been either approved for clinical application or in late-phase clinical trials as blood substitutes, PFC-based nanomaterials may hold great potential in cancer treatment for future clinical translation. However, extensive exposure to PFCs may cause some side effects, including hypotension, cutaneous flushing, fever, pulmonary hypertension, chest tightness, and elevated central venous pressure (Zhou et al., 2016).

### Hydrolysis of Exogenous Peroxide to Produce O_2_

Because the hydrolysis of peroxide will generate O_2_, various peroxides (such as hydrogen peroxide, calcium peroxide, sodium percarbonate, and pyridine endoperoxides) have been utilized as O_2_-producing materials (Harrison et al., 2007; Oh et al., 2009; Wang et al., 2011; Li et al., 2012; Pedraza et al., 2012; Benz et al., 2013). However, the release of O_2_ by the hydrolysis of exogenous peroxide in the absence of a catalyst or trigger was usually slow and limited. It will be more favorable if on-demand and uniform O_2_ delivery to the cells for a sufficiently long time period can be achieved (Liu J. N. et al., 2017). Huang et al. (2016) reported an implantable oxygen-generating depot by coloading CaO_2_ and catalase into the Ca^2+^-crosslinked microencapsulated alginate pellets. Catalase (CAT) in the alginate pellets could catalyze the breakdown of H_2_O_2_ into O_2_, whereas the Ca^2+^-crosslinked alginate matrix could temper the hydrolytic reactivity of CaO_2_/catalase by limiting the infiltration of H_2_O into the pellets, thus prolonging the generation of O_2_. Upon implantation close to the tumor, this *in situ* oxygen-generating depot effectively alleviated the hypoxic regions in tumor and thus resulted in increased chemotherapeutic effect of DOX by promoting ROS production. Liu L. H. et al. (2017) encapsulated CaO_2_ and methylene blue (MB) into liposome to fabricate an O_2_ self-sufficient nanoplatform (LipoMB/CaO_2_) to enhance PDT efficacy in hypoxic tumor. CaO_2_ inside liposomes could react with H_2_O or weak acid to release O_2_ slowly. Upon laser irradiation, ^1^O_2_ activated by the photosensitizer could induce lipid peroxidation to break the liposome and then enlarge the contact area of CaO_2_ with H_2_O, resulting in accelerated O_2_ release.

### Catalytic Decomposition of Intracellular H_2_O_2_ by Utilizing Catalase or Catalase-Like Nanozymes.

Due to the overexpressed H_2_O_2_ in tumor (100 μM–1 mM), various natural enzymes (catalase) and metals or metal-oxide based nanozymes have been applied to overcome tumor hypoxia by catalyzing the *in situ* transformation of endogenous H_2_O_2_ to O_2_. Catalase (CAT) is a catalytic enzyme with a high turnover number to decompose H_2_O_2_ into O_2_ and thus can be employed to relieve tumor hypoxia. However, the nonnegligible disadvantages of CAT, including immunogenicity, the protease-induced degradation, and short half-life, usually restrict its *in vivo* functions after systemic administration. Chemical modification or encapsulating CAT within inorganic or polymer nanostructures has been demonstrated to be an effective approach to overcome these limitations (Chen et al., 2015; Cheng et al., 2016; Zhang R. et al., 2017; Li et al., 2017). Chen et al. (2014) chose PLGA nanoparticles as a carrier to load CAT and platinum anticancer drug. Synergistic release of anticancer drugs and O_2_ triggered by H_2_O_2_ could overcome hypoxia-induced multidrug resistance and thus resulted in improved therapeutic efficacy. By encapsulating CAT into hollow tantalum oxide (TaO_x_), Song et al. obtained a bionanoreactor (TaO_x_@Cat-PEG) combining high-Z element (Ta) and CAT for relieving tumor hypoxia and enhancing RT outcomes. The mesoporous shell of TaO_x_ protected CAT from outside proteases to improve its stability (Song G. S., Chen Y. Y., et al., 2016). Wang H. et al. (2018) reported an *in situ* free radical polymerization method by using a photosensitizer (meso-tetra(p-hydroxyphenyl) porphine (THPP)) as the crosslinker to modify CAT for tumor hypoxia modulation and enhanced PDT. In the obtained CAT-THPP-PEG nanocapsules, the PEG chains polymerized on the surface of CAT could prevent the direct contact between serum proteins and CAT and thus enhanced the enzyme stability, maintained its catalytic activity, and reduced its immunogenicity. Phua et al. (2019) reported that the integration of hyaluronic acid (HA) with CAT could not only improve the physiological stability of the system but also enable active targeting to tumors. The photosensitizer (Ce6)-loaded nanosystem (HA-CAT@aCe6) could target CD44-overexpressed cancer cells, relieve hypoxia by converting endogenous H_2_O_2_ to O_2_, and consequently improve PDT efficacy.

**FIGURE 2:**
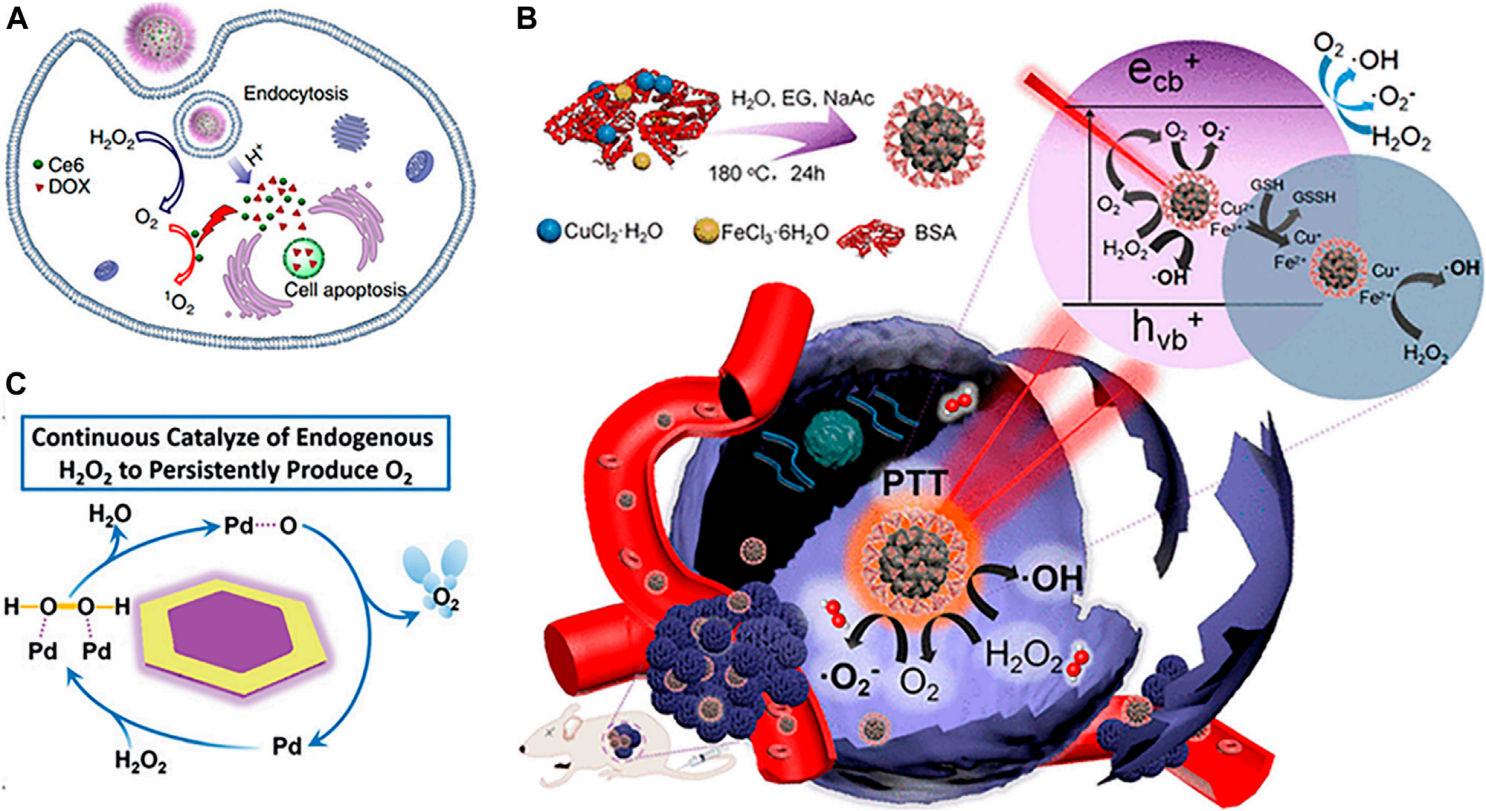
**(A)** Schematic illustration of H-MnO_2_-PEG loaded with DOX and Ce6 for pH-responsive drug delivery and oxygen-elevated PDT (Yang et al., 2017) (Copyright 2017, reproduced with permission from Nature Publishing Group). **(B)** Schematic illustration of synthetic process and therapeutic mechanism of CuFe_2_O_4_ nanospheres (Liu Y. et al., 2018) (Copyright 2018, reproduced with permission from American Chemical Society). **(C)** Schematic illustration of Pd@Au for catalysis of H_2_O_2_ and continuous production of O_2_ (Yang Y. et al., 2019) (Copyright 2016, reproduced with permission from John Wiley and Sons).

Apart from natural enzymes, various nanomaterial-based artificial enzymes show catalase-like activity; one of the typical representatives is MnO_2_. Various MnO_2_ nanostructures have been designed and incorporated into multifunctional nanoplatforms to induce the decomposition of endogenous H_2_O_2_ into O_2_, thus alleviating tumor hypoxia and improving therapeutic efficacy (Prasad et al., 2014; Fan et al., 2015; Abbasi et al., 2016; Yi et al., 2016; Wang Z. et al., 2018). Moreover, MnO_2_ could be decomposed into soluble Mn^2+^ in TME, thus reducing unwanted *in vivo* accumulation and long-term toxicity (Zhu et al., 2016). The released Mn^2+^ could mediate the Fenton-like reaction to convert H_2_O_2_ into the highly reactive •OH, further enhancing the therapeutic potency by introducing extra CDT (Sun et al., 2020). Apart from the abovementioned benefits, MnO_2_ could also be used for drug release, glutathione (GSH) depletion, the regulation of pH, and T1-weighted magnetic resonance (MR) imaging, consequently achieving multimodal theranostic effects and tumor-specific enhanced combination therapy (Fan et al., 2016; Zhang C. et al., 2017; Zhu P. et al., 2018; Zhu H. et al., 2018; Yang G. et al., 2018; Zhang et al., 2019; Pu et al., 2020). For example, Yang et al. (2017) designed an intelligent theranostic platform based on hollow mesoporous MnO_2_ (HMnO_2_) nanoshells for tumor-targeted drug delivery, pH-triggered controllable release, and TME-responsive generation of O_2_ to alleviate tumor hypoxia. Ce6 and DOX were coloaded into HMnO_2_ to achieve combined chemo-photodynamic therapy (Figure 2A). Fluorescence signal of Ce6 and T1-weighted MR signals of the released Mn^2+^ were applied to track the nanoparticles after the injection. Despite great progresses and promising results, the rapid consumption of MnO_2_ during the reaction in TME may restrict its extensive application to a certain extent (Zhang et al., 2018).

Differentiated from the aforementioned self-sacrificing MnO_2_, ferrite materials with catalase-like activity and enhanced stability could be served as a superior candidate for continuous O_2_ supply. For example, Kim et al. (2017) developed continuous O_2_-evolving MnFe_2_O_4_ nanoparticle-anchored mesoporous silica nanoparticles to enhance the PDT effects against hypoxic tumor. The MnFe_2_O_4_ nanoparticles were not consumed during the catalytic reaction and thus could continuously catalyze H_2_O_2_ into O_2_, enabling the subsequent ROS generation from activated photosensitizer Ce6. Yin et al. (2019) reported that MnFe_2_O_4_@MOFs core-shell nanostructure exhibited dual catalytic ability in continuously triggering the decomposition of H_2_O_2_ to release O_2_ and persistently depleting endogenous GSH, resulting in improved PDT. Also, MnFe_2_O_4_ nanoparticles were not consumed during the reaction. Liu Y. et al. (2018) developed CuFe_2_O_4_ nanospheres that integrated PDT, PTT, photoenhanced CDT, and MR imaging functions along with TME-modulating capacity. The CuFe_2_O_4_ nanospheres regulated the TME through the decomposition of H_2_O_2_ to O_2_ and the depletion of GSH, which relieved the tumor hypoxia and antioxidant capability, thus further improving the photoenhanced CDT and PDT efficiency (Figure 2B).

Various Fe-doped nanoplatforms have been reported to catalyze the conversion of endogenous H_2_O_2_ to O_2_ and thus could enhance the therapeutic effects against hypoxic tumor, including Fe-doped polydiaminopyridine nanofusiforms (Fe-PDAP) (Bai et al., 2018), Fe^III^ doped C_3_N_4_ nanosheets (Ma et al., 2016), and Fe^3+^-driven assembly of fluorenylmethyloxycarbonyl (Fmoc) protected amino acids (Fmoc-Cys/Fe) (Li Y. et al., 2020). Lan et al. (2018) developed a nanoscale MOF (Fe-TBP, constructed from Fe_3_O clusters and 5,10,15,20-tetra(*p*-benzoato)porphyrin (TPB)) as a nanophotosensitizer to overcome tumor hypoxia for PDT-primed cancer immunotherapy. Intracellular H_2_O_2_ could be decomposed by the Fe_3_O clusters to generate O_2_ through a Fenton-like reaction, whereas the produced O_2_ was converted to cytotoxic singlet oxygen (^1^O_2_) by photoexcited porphyrins. Prussian blue (PB), a clinical medicine approved by U.S. FDA for the treatment of radioactive exposure, has been proven with catalase-like activity (Cai et al., 2016; Zhou et al., 2018). Yang Z. L. et al. (2018) fabricated a PB-based integrated nanoplatform to elevate O_2_ and ROS for highly efficient PDT.

Other noble metals or metal oxide–based nanozymes with catalase-like activity have also been applied to overcome tumor hypoxia via H_2_O_2_-activated catalytic O_2_ generation, thereby augmenting effect of ROS-generated cancer therapy, such as CeO_2_ (Dong et al., 2020), RuO_2_ (Huang et al., 2020; Xu et al., 2020), V_2_O_5_ (Li C. et al., 2020), mesoporous manganese cobalt oxide derived from MOFs (Wang et al., 2019), Pd@Pt nanoplates (Wei et al., 2018), gold nanoclusters (Liu, C. P., et al., 2017), MOF–Au nanohybrid (He et al., 2019), Pt nanoparticles decorated on MOFs (Zhang et al., 2018), Pt-based core-shell nanoplatform (Wang X. S. et al., 2018), two-dimensional Pd@Au bimetallic core-shell nanostructure (Yang Y. et al., 2019), etc. By taking the advantage of dual enzyme-mimic catalytic activity of ultrasmall CeO_2_, Dong et al. (2020) fabricated a nanocomposite with hyperthermia-enhanced peroxidase-like activity, catalase-mimic activity, and GSH depletion for efficient tumor therapy in the NIR-II window. Huang et al. (2020) reported that a multifunctional artificial metalloprotein nanoanalogue, RuO_2_-hybridized ovalbumin (OVA) nanoanalogues, not only exhibited photothermal/photodynamic effect under NIR light irradiation but also effectively alleviated tumor hypoxia via catalysis of intracellular H_2_O_2_ to produce O_2_, thereby concurrently enhancing PDT and reversing the immunosuppressive TME. Yang B. et al. (2019) reported a two-dimensional Pd@Au core-shell nanostructure (TPAN) that could continuously catalyze endogenous H_2_O_2_ to generate O_2_ for relieving tumor hypoxia to overcome hypoxia-induced RT resistance. Moreover, the catalytic activity of TPAN toward H_2_O_2_ could be enhanced via the surface plasmon resonance effect triggered by NIR-II laser irradiation (Figure 2C). Wei et al. (2018) reported that Pd@Pt-PEG-Ce6 nanocomposite could not only deliver photosensitizers to tumor sites but also trigger the decomposition of endogenous H_2_O_2_ to produce O_2_ for a long period of time. Moreover, the moderate photothermal effect of Pd@Pt-PEG-Ce6 under 808 nm laser irradiation accelerated its catalytic decomposition of H_2_O_2_ to O_2_. Liu C. P. et al. (2017) reported that the amine-terminated, PAMAM dendrimer-encapsulated gold nanoclusters (AuNCs-NH_2_) can produce O_2_ to improve PDT via the catalase-like activity. Importantly, AuNCs-NH_2_ exhibited the catalase-like activity over a broad pH range (pH 4.8–7.4).

### Generating O_2_ by Water-Splitting Photocatalysts

Compared to the limited intracellular concentration of H_2_O_2_, H_2_O is the most abundant compound in living organisms. Consequently, using H_2_O as an alternative O_2_-generating reactant, the water-splitting strategy could provide unlimited raw materials for *in vivo* O_2_ release. As a typical paradigm, Zheng et al. (2016) reported the use of carbon-dot-decorated C_3_N_4_ nanocomposite as a water-splitting catalyst to produce O_2_ to overcome tumor hypoxia and improve the PDT effect. The carbon dots were doped to decrease the band gap of C_3_N_4_, and a 630 nm laser was applied as the trigger to induce the water splitting. Chen et al. (2020) reported that *in situ* photocatalysis of TiO porphyrin encapsulated in folate liposome could not only conquer tumor hypoxia but also generate sufficient ROS to suppress the tumor growth. Analogous to the aforementioned photocatalysts, the photosensitizer nanoparticle-loaded photosynthetic bacteria were developed for tumor-targeted photosensitizer (indocyanine green, ICG) delivery and *in situ* photocatalyzed O_2_ generation. This biomimetic system combined the photosynthetic capability of *Synechococcus* 7942 (a natural photosynthetic cyanobacterium) and the theranostic effect of ICG-encapsulated human serum albumin nanoparticles (Liu et al., 2020). Since hypoxic tumors are usually located in the deep tissues, the penetration depth of the laser is a limitation.

## Conclusion and Challenges

We herein present an overview of current strategies to overcome the tumor hypoxia in ROS-generated cancer therapy. Despite great progresses and promising results, most attempts still remain at early stages of development. These strategies suffer from some disadvantages, for example, side effects after intravenous injection, H_2_O_2_ dependence in H_2_O_2_-mediated O_2_ production, rapid consumption or easy inactivation/instability of natural enzyme and nanozymes, and poor light penetration in photoactivated O_2_ production. Moreover, to achieve enhanced therapeutic efficacy, integration of multiple therapeutic/diagnostic capability and oxygen-supply ability into one nanosystem has become the most commonly used strategy to treat hypoxic tumors. Consequently, complicated and tedious preparation procedures are usually needed. To maximize their capabilities and minimize the side effects, toxicity and immunogenicity of all the involved components should be comprehensively evaluated before clinical trials. In addition, the degradability of the materials should be guaranteed, which will enable the body to clear them after performing the designated pharmacological functions.
